# Alpha Particles Induce Autophagy in Multiple Myeloma Cells

**DOI:** 10.3389/fmed.2015.00074

**Published:** 2015-10-19

**Authors:** Jean-Baptiste Gorin, Sébastien Gouard, Jérémie Ménager, Alfred Morgenstern, Frank Bruchertseifer, Alain Faivre-Chauvet, Yannick Guilloux, Michel Chérel, François Davodeau, Joëlle Gaschet

**Affiliations:** ^1^CRCNA – UMR 892 INSERM, Nantes, France; ^2^6299 CNRS, Nantes, France; ^3^Université de Nantes, Nantes, France; ^4^Institute for Transuranium Elements, Karlsruhe, Germany; ^5^Nuclear Medicine Department, CHU Nantes, Nantes, France; ^6^Institut de Cancérologie de l’Ouest, Saint-Herblain, France

**Keywords:** bismuth-213, multiple myeloma, radiobiology, autophagy, immunogenicity, ionizing radiation

## Abstract

**Objectives:**

Radiation emitted by the radionuclides in radioimmunotherapy (RIT) approaches induce direct killing of the targeted cells as well as indirect killing through the bystander effect. Our research group is dedicated to the development of α-RIT, i.e., RIT using α-particles especially for the treatment of multiple myeloma (MM). γ-irradiation and β-irradiation have been shown to trigger apoptosis in tumor cells. Cell death mode induced by ^213^Bi α-irradiation appears more controversial. We therefore decided to investigate the effects of ^213^Bi on MM cell radiobiology, notably cell death mechanisms as well as tumor cell immunogenicity after irradiation.

**Methods:**

Murine 5T33 and human LP-1 MM cell lines were used to study the effects of such α-particles. We first examined the effects of ^213^Bi on proliferation rate, double-strand DNA breaks, cell cycle, and cell death. Then, we investigated autophagy after ^213^Bi irradiation. Finally, a coculture of dendritic cells (DCs) with irradiated tumor cells or their culture media was performed to test whether it would induce DC activation.

**Results:**

We showed that ^213^Bi induces DNA double-strand breaks, cell cycle arrest, and autophagy in both cell lines, but we detected only slight levels of early apoptosis within the 120 h following irradiation in 5T33 and LP-1. Inhibition of autophagy prevented ^213^Bi-induced inhibition of proliferation in LP-1 suggesting that this mechanism is involved in cell death after irradiation. We then assessed the immunogenicity of irradiated cells and found that irradiated LP-1 can activate DC through the secretion of soluble factor(s); however, no increase in membrane or extracellular expression of danger-associated molecular patterns was observed after irradiation.

**Conclusion:**

This study demonstrates that ^213^Bi induces mainly necrosis in MM cells, low levels of apoptosis, and autophagy that might be involved in tumor cell death.

## Introduction

Ionizing radiation (IR) is widely used in the treatment of cancer. External beam radiation therapy (EBRT) using X- or γ-rays is a primary treatment for a wide range of localized cancer, brachytherapy is commonly used as an effective treatment for cervical, prostate, breast, and skin cancer, and radioimmunotherapy (RIT) using β-emitters has proved its efficacy for the treatment of refractory non-Hodgkin lymphoma (1, 2).

New trials are now assessing the efficacy of α-particle emitters in non-targeted or targeted therapies against hematological or solid cancers (3). Indeed, the physical and biological characteristics of those radioisotopes make them attractive candidates for the treatment of disseminated or residual cancers. α-particles exhibit a high linear energy transfer (LET) (~100 keV/μm) with a short range of 50–90 μm into the tissues combined with a high energy of 5–9 MeV. In addition, energy deposition along their path follows a Bragg peak, resulting in an increased LET at the end of the track and therefore an increased cytotoxicity. Like other high LET particles, α-emitters induce complex clusters of DNA damages including DNA double-strand breaks (DSBs) which lead to a cell cycle arrest that is more marked than with γ-rays (4). Cell nucleus traversal with one to three α-particles is sufficient to cause tumor cell death when 1000–5000 β-emitters would be required (5). Furthermore, radiobiological effects associated with α-radionuclides are largely independent of dose rate, oxygenation, and cell proliferation (6). A few studies have investigated the cell death mechanisms *in vitro* and *ex vivo* after α-irradiation and led to contrasting results. Some groups showed that cells undergo apoptosis following exposure to ^213^Bi (7–9) while others observed cell death independent of apoptosis (10–12), therefore reinforcing the need for further investigation of such mechanisms.

Diverse α-emitters have been used in the clinic so far, displaying short half-lives, like ^213^Bi, ^211^At, and ^212^Pb as well as long-lived like ^223^Ra and ^225^Ac (3). Our group has done several *in vitro* and preclinical studies on multiple myeloma (MM) (12–16) using ^213^Bi produced by ^225^Ac/^213^Bi radionuclide generators. Therefore, we thought to further investigate the impact of this α-emitter on the radiobiology of MM cells, especially cell death mechanisms.

Moreover, experiments using EBRT have shown that in addition to direct tumor cell killing, IR can generate specific immune responses directed against tumor cells. Besides creating a local inflammatory context, it has been demonstrated that irradiation can induce immunogenic cell death (ICD) of cancer cells along with the release of danger-associated molecular patterns (DAMPs) (17, 18). Inflammation, ICD, and DAMPs promote the recruitment of immune cells to the tumor site, such as dendritic cells (DCs), which can internalize dying tumor cells. Then cross-presentation of tumor antigens by activated DCs primes antitumor T-cell response (19). Recently, we and others have shown that α-particle emitters ^213^Bi or ^224^Ra can induce similar ICD of tumor cells (20–22) in combination with Hsp70 and HMGB-1 release, leading to efficient T-cell-dependent antitumor response (20, 21).

The aim of this study was to investigate the radiobiological effects, in particular cell death mechanisms, of ^213^Bi on MM cells and to assess if irradiation of these tumor cells can lead to immune cell activation. Murine 5T33 and human LP-1 MM cell lines were used; we showed that ^213^Bi induces inhibition of proliferation, DSBs, cell cycle arrest, and autophagy in both cell lines. Inhibition of autophagy prevented ^213^Bi-induced inhibition of proliferation in LP-1, suggesting that autophagy is one of the tumor cell death mechanisms after α-irradiation. We then evaluated the immunogenicity of irradiated cells and found that irradiated LP-1 can activate DCs through the secretion of soluble factor(s).

## Materials and Methods

### Cell Culture, ^213^Bi-Irradiation, and Pharmacological Treatment

5T33 (provided by Dr. Radl, TNO Institute, Leiden, Netherlands) and LP-1 cells (DSMZ: ACC 41) were maintained in RPMI 1640 (Gibco) supplemented with 10% FCS, 2 mM glutamine (Gibco), 100 U/mL penicillin, and 100 μg/mL streptomycin (Gibco) at 37°C and 5% CO_2_.

At least 2 h prior to irradiation, the cells were plated at 8 × 10^5^ cells/mL in fresh culture medium. A solution containing ^213^Bi diluted in culture medium was then added to the cells. Thus, a final concentration of 4 × 10^5^ cells/mL was obtained in the presence of the desired activity of ^213^Bi.

For autophagy inhibition, cells were treated with 1.25 mM 3-methyladenine (3-MA) (Sigma).

### Preparation of ^213^Bi-BSA

Cyclohexyl diethylene triamine penta-acetic acid (CHX-A″-DTPA; Macrocyclics) was conjugated to BSA (Sigma) and controlled by indium labeling. For labeling with ^213^Bi, conjugated BSA was incubated with ^213^Bi eluted from a ^225^Ac/^213^Bi generator (Institute for Transuranium Elements, Karlsruhe, Germany) for 10 min at 37°C in 0.4 M ammonium acetate (pH, 5.3). The resulting ^213^Bi–BSA conjugate was purified from unbound ^213^Bi by size exclusion chromatography using a PD-10 column (GE Healthcare).

### ^3^H-Thymidine Incorporation Assay

Approximately 16 h after ^213^Bi-irradiation, cells were plated in quadruplicates at 4 × 10^5^ cells/mL in 100 μL in 96-well flat-bottom microtiter plates and incubated at 37°C. Forty-two hours after irradiation, 10 μL of ^3^H-thymidine (925 kBq/mL; Perkin Elmer) was added to each well and incubated at 37°C. Six hours later (i.e., 48 h after irradiation), cells were harvested (Harvester 96 – Tomtec) on glass fiber Filtermat A (Perkin Elmer). Radioactive emission was amplified with Betaplate Scint (Perkin Elmer) and read using 1450 Microbeta Plus counter (Wallac). Results are expressed as quadruplicate mean ± SD.

### Colony-Forming Assay

Sixteen hours after irradiation, cells were plated in 96-well U-bottom microtiter plates at densities of 30, 10, 1, and 0.3 cells per well and cultured 2–3 weeks at 37°C, 5% CO_2_. Growth frequency was then calculated using Poisson distribution.

### γH2AX Staining

5T33 and LP-1 cells were fixed with 2% PFA (EMS, Washington, PA, USA) and permeabilized with methanol in PBS–0.5% BSA. γH2AX staining was performed with PE-γH2AX antibody (BD Pharmingen) for 1 h at 4°C. After two washes with PBS–0.5% BSA and once with PBS, analysis of at least 10,000 events was performed using a BD FACScalibur flow cytometer and FlowJo software.

### Cell Cycle Analysis

Cells were harvested, centrifuged, and resuspended in 200 μL PBS. LP-1 and 5T33 were fixed by adding 1 mL of cold ethanol or methanol, respectively, for at least 10 min at 4°C. Cells were then stained with propidium iodide as described previously (13) and at least 10,000 events were analyzed using a BD FACScalibur flow cytometer and FlowJo software.

### Annexin V and 7-AAD Staining

Staining was performed according to manufacturer’s instructions (BD Annexin V-PE Apoptosis detection kit). Briefly, 10^5^ cells were stained with 5 μL Annexin V-PE and/or 7-AAD for 15 min at room temperature in the dark. At least 10,000 events were analyzed using BD FACScalibur flow cytometer and FlowJo software.

### Western Blotting

Cells were lysed in 1% Triton X-100 lysis buffer containing protease inhibitors (5 μg/mL aprotinin, 5 μg/mL leupeptin, and 4 mM Pefablock^®^) for 15 min on ice. Cell debris were pelleted at 11,000 × *g* for 20 min at 4°C, and protein concentrations were determined by BCA reagent (Interchim). Proteins were separated by SDS-PAGE in a 15% acrylamide gel and transferred onto PVDF membranes. Blots were incubated 1 h in blocking buffer (TBS, 0.1% Tween 20, and 5% milk) before incubation for 2 h with primary antibodies either rabbit anti-LC3B antibody (1 μg/mL; Sigma) that recognizes both LC3B-I and LC3B-II forms or goat anti-actin (C-11, 50 ng/mL; Santa Cruz, CA, USA). Blots were then incubated 1 h with the appropriate HRP-conjugated secondary antibody and processed to detect electrochemiluminescence (Roche). The signal was acquired with the Fusion FX7 camera and its intensity determined using Bio-1D software (Vilber Lourmat).

### Transmission Electron Microscopy

Non-irradiated and ^213^Bi-irradiated 5T33 and LP-1 cells were fixed in cacodylate buffered 4% glutaraldehyde for 15 min at 4°C, washed, and post-fixed in cacodylate buffered 2% osmium tetroxide for 20 min at 4°C. Samples were dehydrated in successive dilutions of ethanol and embedded overnight in Epon at 37°C and for two other days at 55°C. Sections (80 nm thick) were cut with an Ultracut E ultramicrotome (Reichert-Jung), mounted on copper grids, stained with uranyl acetate, and observed on a JEOL 1010 TEM.

### DCs Production

Human DCs were obtained by differentiation of human monocytes. Monocytes were purified from PBMCs by counterflow centrifugal elutriation and cultured 5 days in RPMI supplemented with 2% human albumin, 2 mM glutamine, 100 U/mL penicillin, 100 μg/mL streptomycin, GM-CSF 1000 IU/mL (CellGro), and IL-4 200 IU/mL (BruCells). Mouse DCs were obtained by differentiation of bone marrow from C57Bl/6 thigh bone as described previously (20).

### Coculture Assays

For DC coculture assay with tumor cells, five million ^213^Bi-treated tumor cells (LP-1 or 5T33) or non-irradiated tumor cells were centrifuged 48 h after irradiation. Tumor cell pellets were resuspended in 1 mL fresh medium and added to one million DCs (human or mouse) in a total volume of 2 mL and plated in six-well dishes. Twenty-four hours after coculture at 37°C, DC maturation was analyzed by immunofluorescence phenotyping.

For DC coculture assay with tumor cell supernatant, 48 h after irradiation, 1 mL of tumor cell supernatant, obtained after centrifugation of five million irradiated or non-irradiated tumor cells (LP-1 or 5T33), was added to one million DCs (human or mouse) in a total volume of 2 mL and plated in 12-well plates.

### Immunofluorescence Analysis

Cells were washed once in 0.1% BSA–PBS and then stained for 1 h at 4°C with primary antibody. When secondary antibody was needed, cells were washed three times in 0.1% BSA–PBS before incubation with secondary antibody. After staining, cells were washed twice in 0.1% BSA–PBS and once in PBS before acquisition in flow cytometer. The following antibodies and their respective control isotypes were used in this study: PE anti-human CD80 (L307.4; BD), APC anti-human CD83 (HB15e; BD), APC anti-human CD86 (2331 FUN-1; BD), and PE anti-HLA-DR (G46.6; BD). All immunofluorescence analyses were performed using a FACScalibur flow cytometer (BD Pharmingen) and analyzed with FlowJo software.

## Results

### ^213^Bi Irradiation Hampers Cell Proliferation

**Figure 1:**
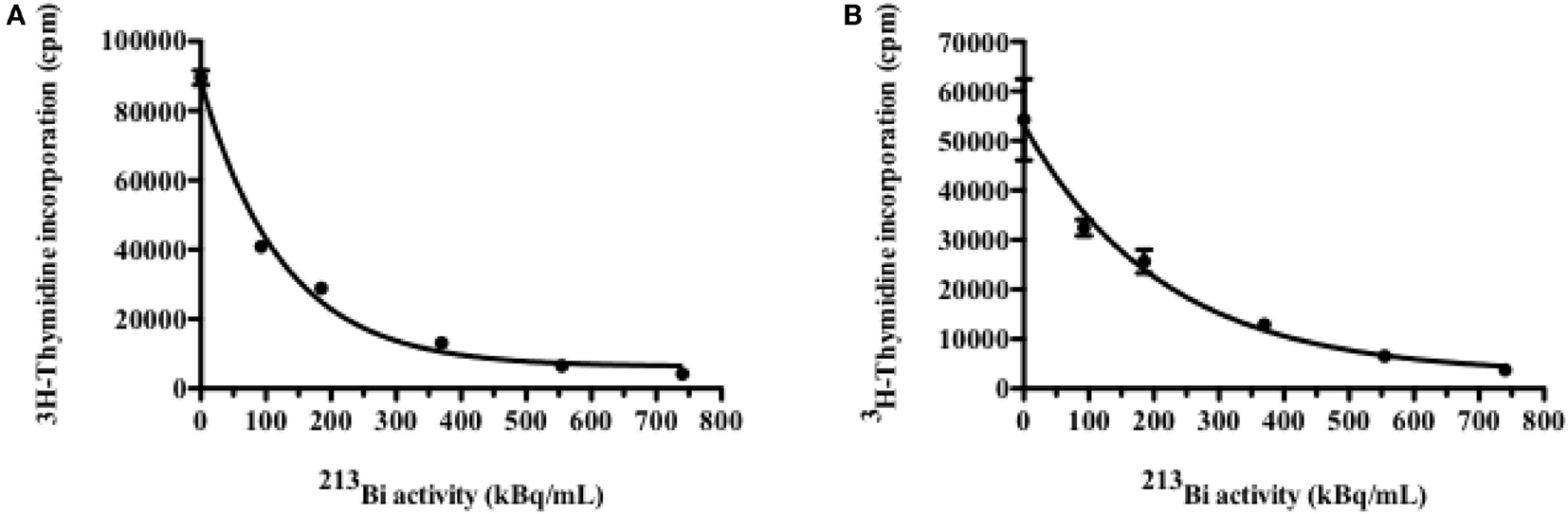
**Inhibition of cell proliferation by ^213^Bi irradiation**. Proliferation of irradiated 5T33 **(A)** and LP-1 **(B)** cells assessed 48 h after irradiation by incorporation of ^3^H-thymidine. Data are expressed as quadruplicate mean ± SD and are representative of two independent experiments.

5T33 cells and LP-1 cells were irradiated with increasing activities of ^213^Bi and their proliferation rate was assessed 48 h later by ^3^H-thymidine incorporation assay. Results show a dose-dependent inhibition of ^3^H-thymidine incorporation attesting of a diminution of DNA synthesis and as a result a diminution of their proliferation ability (Figure 1). ^213^Bi activities sufficient to cause at least 80% inhibition of proliferation for each cell line were then used for the rest of the study, respectively, 370 kBq/mL for 5T33 and 425 kBq/mL for LP-1. Such activities ensure a strong biological effect but remain low enough that the cells are not sterilized by irradiation.

### Inhibition of Proliferation is Correlated with Tumor Cell Death

**Figure 2:**
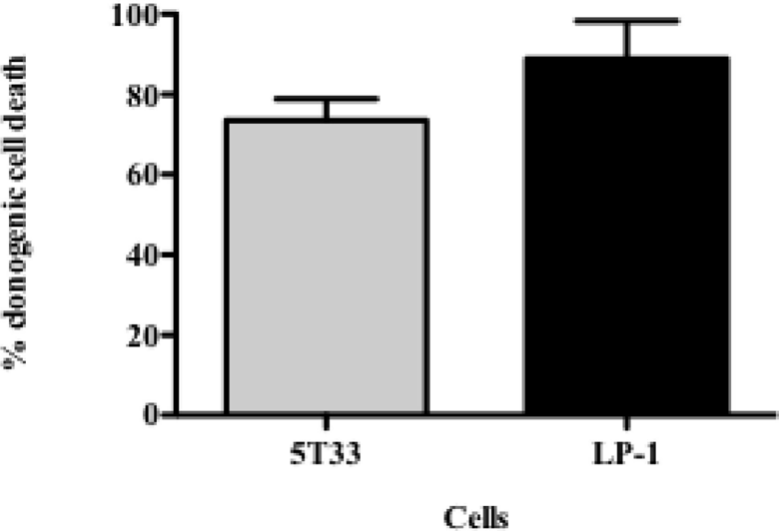
**Clonogenic death of 5T33 and LP-1 cells after ^213^Bi irradiation**. Cell death of ^213^Bi-irradiated 5T33 (370 kBq/mL) and LP-1 (425 kBq/mL) was calculated as defined in Table 1. Data represent mean ± SD of three independent experiments.

In order to determine to what extend the inhibition of proliferation had an impact on tumor cell survival, we performed colony-forming assays. This approach consists in seeding irradiated and non-irradiated cells in clonal conditions to measure the impact of ^213^Bi irradiation on cell growth efficiency. We observed that clonogenic cell death after irradiation reached 73.5 ± 4.6% for 5T33 cells treated at 370 kBq/mL and 88.8 ± 7% for LP-1 treated at 425 kBq/mL (Figure 2; Table 1). This confirmed that blockade of 5T33 and LP-1 cell proliferation is indeed correlated to the same extent to tumor cell death induced on the long term after ^213^Bi treatment.

**Table 1:** **Colony-forming assay of 5T33 and LP-1 cells after ^213^Bi irradiation**.

Cells	Condition	Growth efficiency (%)	Survival (%)	Death (%)
5T33	Control	22.4 ± 6.0	100	0
	370 kBq/mL	5.8 ± 1.8	26.5 ± 4.6	73.5 ± 4.6
LP-1	Control	40.2 ± 31.6	100	0
	425 kBq/mL	6.4 ± 6.7	11.2 ± 7.1	88.8 ± 7.1

*Colony-forming assays were performed 16 h after irradiation with non-irradiated or ^213^Bi-irradiated 5T33 (370 kBq/mL) and LP-1 (425 kBq/mL) cells to determine growth using Poisson distribution. Data represent mean ± SD of three independent experiments*.

### ^213^Bi Induces DNA DSBs in MM Cells

**Figure 3:**
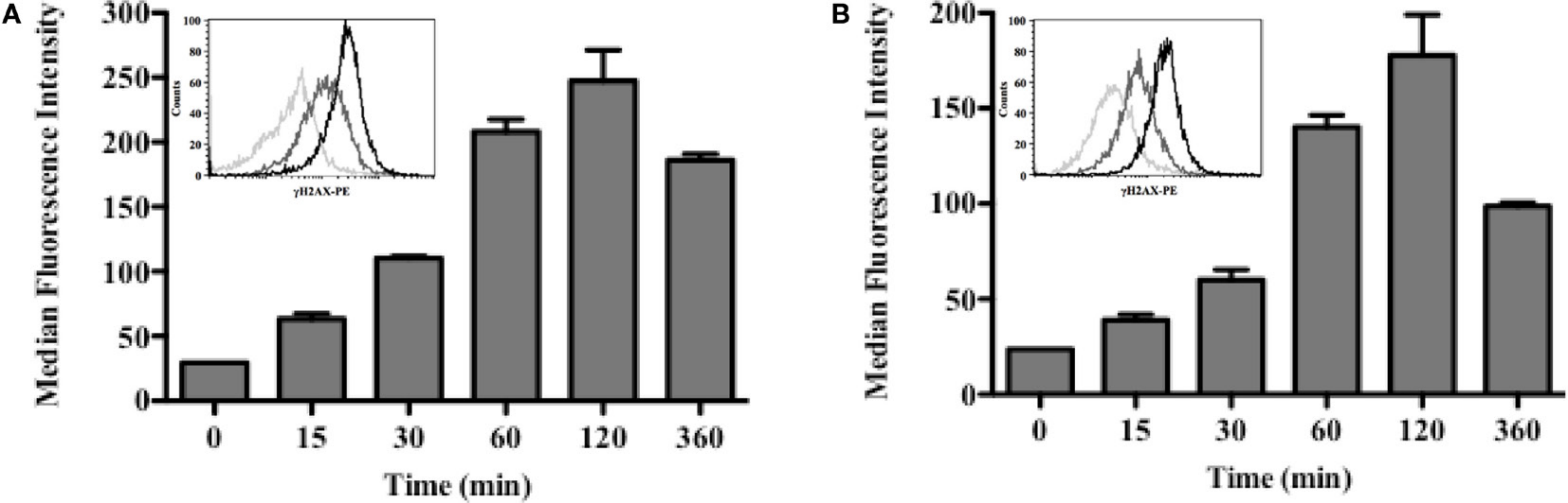
**DSBs are induced shortly after irradiation with ^213^Bi**. 5T33 **(A)** and LP-1 **(B)** cells were irradiated at 370 and 425 kBq/mL, respectively. DSBs were analyzed at different time points after irradiation by flow cytometry using an anti-γH2AX mAb. Flow cytometry histograms illustrate homogeneous γH2AX staining for both cell lines: 0 min (light gray), 30 min (dark gray), and 120 min (black). Bar graphs represent median ± SD of two independent experiments.

Induction of DSBs in 5T33 and LP-1 cells was investigated after ^213^Bi irradiation at 370 kBq/mL or 425 kBq/mL, respectively (Figure 3). Irradiation appeared homogenous since for both cell lines, the entire cell population exhibited γH2AX staining (Figure 3). For both cell lines also, DSBs were detectable already 15 min after treatment and their amount increased following the same kinetic over time to reach a maximum at 2 h. Then, phosphorylation of H2AX histone decreased, as observed 3 h after irradiation.

### ^213^Bi Blocks Cell Cycle

**Figure 4:**
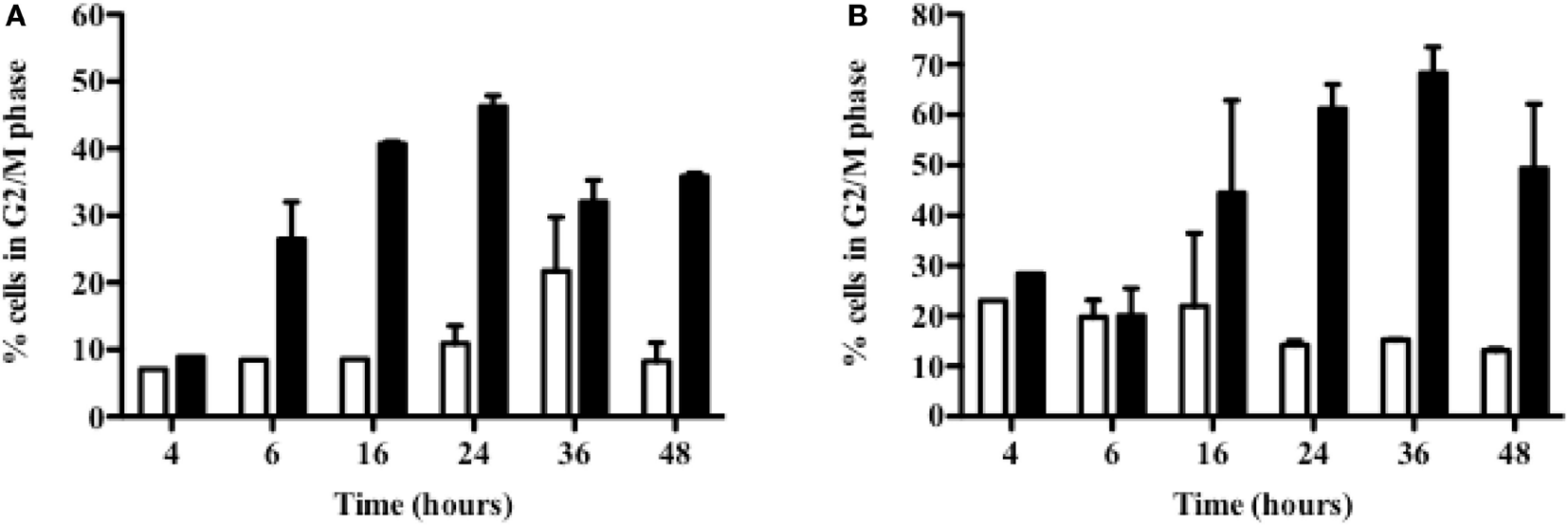
**Accumulation in G2 phase of ^213^Bi-irradiated 5T33 and LP-1 cells**. 5T33 **(A)** and LP-1 **(B)** cells were irradiated at 370 and 425 kBq/mL, respectively. Then cell cycle analysis was performed at different time points after ^213^Bi irradiation of the cells (black bars) and compared to control non-irradiated cells (white bars). Data represent mean ± SD of two independent experiments.

Cell cycle arrest can arise from DNA damages; therefore, the effect of ^213^Bi on 5T33 and LP-1 cell cycle at different time points following irradiation was then investigated (Figure 4). Accumulation in the G2/M phase occurred quite rapidly after irradiation in both cell lines. In 5T33 cells (Figure 4A), cell cycle blockade was seen as soon as 6 h after irradiation with 370 kBq/mL of ^213^Bi and reached a maximum at 24 h with around 45% of the cells in the G2 phase. In LP-1 cells (Figure 4B), cell cycle blockade was slower, being noticeable 24 h after treatment with 425 kBq/mL and reaching a maximum at 36 h with almost 63% of the cells blocked in the G2 phase. These results demonstrate that α-particles cause a blockade at the G2/M checkpoint with a kinetic that varies depending on the cell line.

### ^213^Bi Induces Necrosis in MM Cells

**Figure 5:**
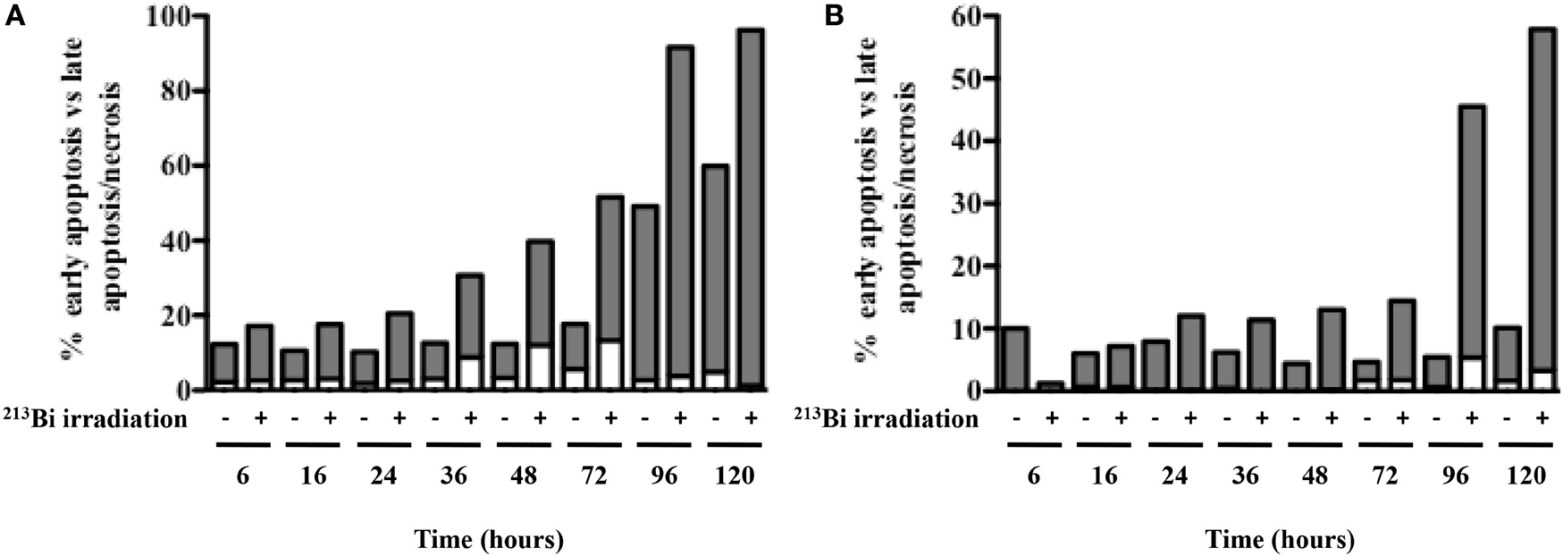
**Apoptosis and necrosis in MM cells after ^213^Bi irradiation**. Early apoptosis (white bars) and late apoptosis/necrosis (gray bars) were assessed at different time points after irradiation of 5T33 cells **(A)** at 370 kBq/mL and LP-1 cells **(B)** at 425 kBq/mL. Data are representative of two independent experiments.

Since 5T33 and LP-1 cells experience serious DSBs, are blocked in G2 phase, and stop proliferating after ^213^Bi irradiation, experiments were conducted to determine the mechanisms that would eventually lead to cell death. Annexin V/7AAD staining was performed on the two MM cell lines at different time points after irradiation to investigate early apoptosis [Annexin V (+) cells] as well as late apoptosis and necrosis [Annexin V (+) 7AAD (+) cells] (Figure 5). Non-irradiated MM cells were studied in parallel. Early apoptosis was barely detectable in any of the cell lines and was not observed immediately after irradiation but to a small extent at later time point, after 36 h for 5T33 and 96 h for LP-1. These data suggest that cell death occurs mainly through necrosis. This was confirmed by active caspase-3 detection and DNA fragmentation analysis that also showed small levels of early apoptosis (data not shown). In 5T33 cells, necrosis appeared at 36 h and reached a maximum at 96 h after irradiation at 370 kBq/mL of ^213^Bi. In LP-1, necrosis seems to arise later and is strikingly increased at 96 h after irradiation at 425 kBq/mL. Non-irradiated 5T33 control cells kept dividing actively over the time of the experiment and despite splitting exhibited important cell death as observed after 96 h of culture.

### ^213^Bi Induces Autophagy in MM Cells

Autophagy can be induced in cells as a response to a variety of stress factors, including IR-induced oxidative stress, and is characterized by an increased number of autophagosomes that exhibit a lipid bilayer membrane. At the molecular level, when autophagy is activated, the LC3-B protein is converted from the cytosolic LC3B-I form to the LC3B-II form recruited on the autophagosome membrane. Common methods to assess autophagy are Western blot of the LC3-B protein, TEM, and use of autophagy inhibitors.

**Figure 6:**
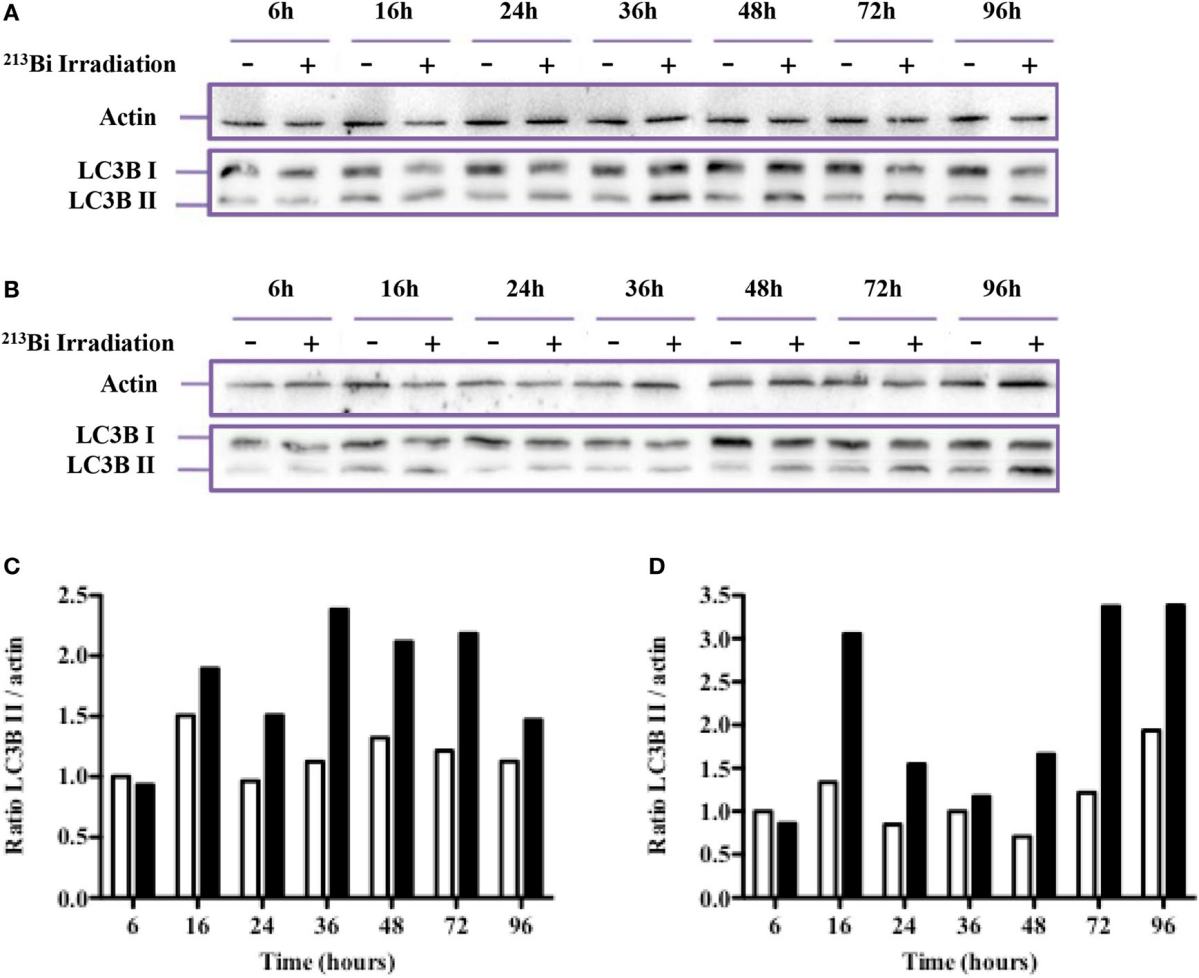
**^213^Bi irradiation induces autophagy in 5T33 and LP-1 cells**. To analyze autophagy, Western blot analysis of LC3B I conversion to the LC3B II form was performed at different time points after irradiation in 5T33 cells **(A)** at 370 kBq/mL and LP-1 cells **(B)** at 425 kBq/mL. Densitometry analysis of the blots was performed to determine the LC3B II to actin ratios in control non-irradiated (white bars) and irradiated (black bars) 5T33 **(C)** and LP-1 **(D)** cells. Data are representative of three independent experiments.

**Figure 7:**
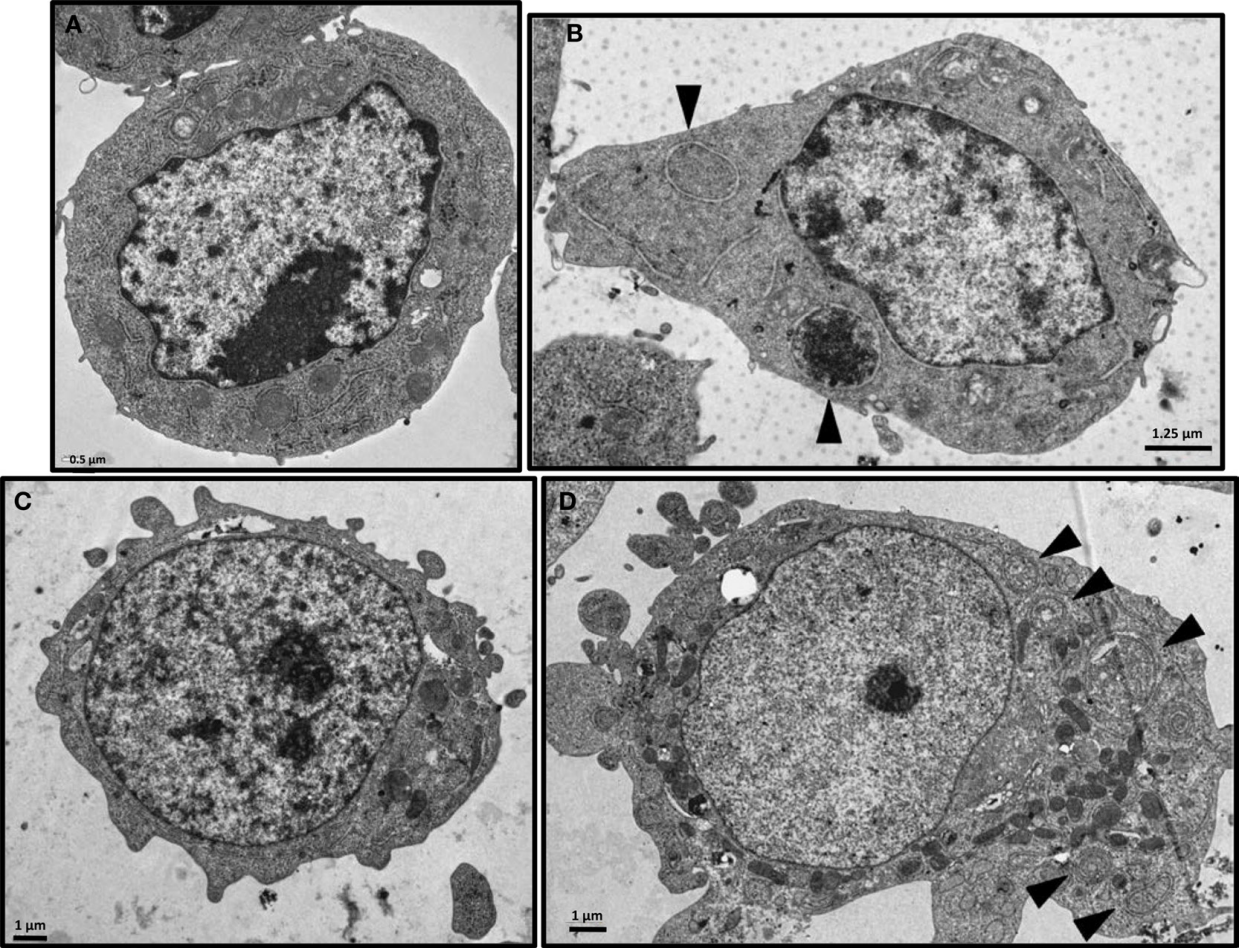
**Electron microscopy of ^213^Bi-treated cells**. Analysis was performed on 5T33 cells: untreated **(A)** or 48 h after irradiation with 370 kBq/mL ^213^Bi **(B)** and on LP-1 cells: untreated **(C)** or 72 h after irradiation with 425 kBq/mL ^213^Bi **(D)**. Autophagic vacuoles presenting lipid bilayer membrane are indicated by arrows.

Western blot assays on irradiated and non-irradiated MM cell lysates were analyzed by standardization of LC3B-II form to actin as mentioned by Mizushima and Yoshimori (23) and showed a strong induction of LC3B-I form conversion to the LC3B-II form after ^213^Bi irradiation (Figure 6). Accumulation of the LC3B-II form of the LC3B protein was observed in 5T33 cells at 24 h, further increased at 36 h, and remained stable until 72 h after irradiation at 370 kBq/mL. In LP-1 cells, after a transient and early accumulation at 16 h, LC3B-II form decreased before raising again at 48 h and remained stable at 96 h after treatment with 425 kBq/mL of ^213^Bi. For both cell lines, non-irradiated cells did not exhibit major changes in LC3B-II form accumulation over the time of experiments (Figure 6). Moreover, the presence of autophagosomes in ^213^Bi-treated 5T33 and LP-1 cells was confirmed by TEM analysis (Figure 7). Altogether, these results demonstrate that α-particles induce autophagy in MM cells.

**Figure 8:**
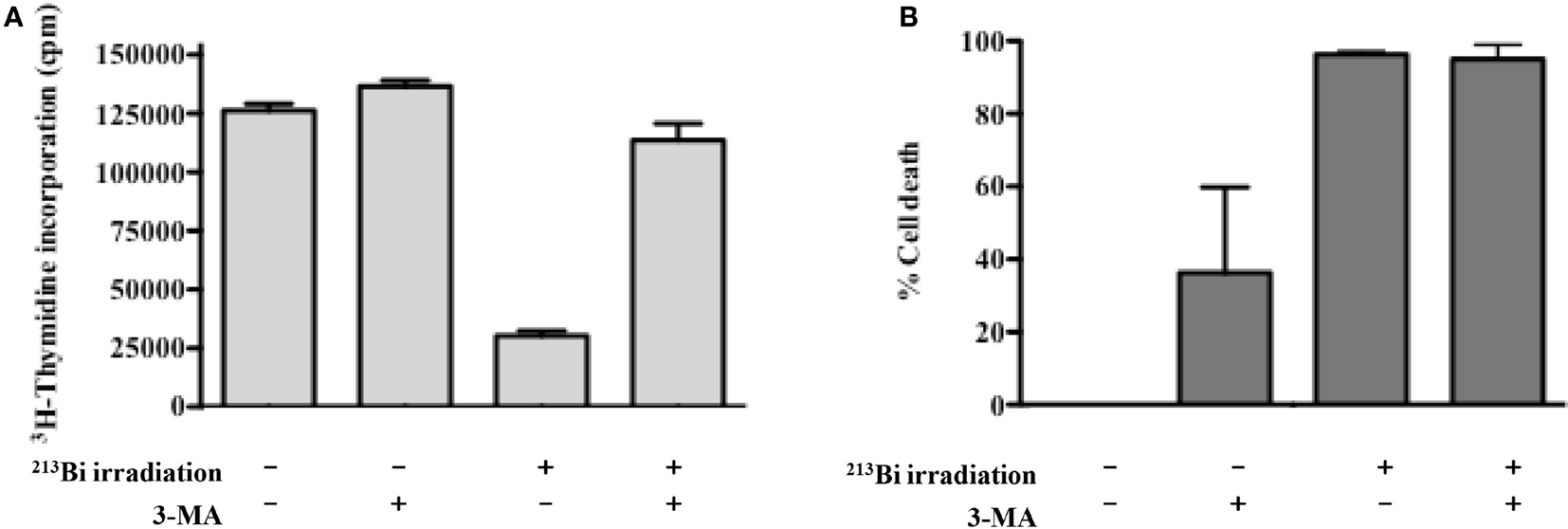
**Inhibition of autophagy restores proliferation of LP-1 cells but do not impact clonogenic cell death**. **(A)** Proliferation of irradiated or control LP-1 cells with or without autophagic inhibitor was assessed 72 h after irradiation by incorporation of ^3^H-thymidine. **(B)** Clonogenic cell death of untreated LP-1 or after ^213^Bi irradiation with or without autophagic inhibitor. Data represent mean ± SEM of two independent experiments.

Autophagy has been described both as a cell survival and cell death mechanism depending on the stimuli, context, and/or cell lines studied. To assess the role of autophagy in our cell lines after irradiation with ^213^Bi, both proliferation and colony-forming assays were conducted in the presence of an autophagy ­inhibitor – 3-methyladenine (3-MA) – which inhibits more specifically class III phosphoinositide 3-kinase activity known to be essential for autophagy induction. In 5T33, at 1.25 mM of 3-MA, the proliferation rate after ^213^Bi treatment with or without inhibition of autophagy was quite similar (data not shown). However, for LP-1 cells, when autophagy was blocked by 3-MA, the proliferation rate after irradiation was affected. As shown in Figure 8A, the amount of 3-MA used in that experiment had no major effect on proliferation of non-irradiated LP-1 cells. As shown previously, treatment of LP-1 cells at 425 kBq/mL with ^213^Bi induced around 90% decrease in proliferation rate. Then, when LP-1 cells were irradiated with ^213^Bi in the presence of 3-MA, the cells appeared protected from irradiation and kept proliferating at least for 72 h. These results raise the hypothesis that autophagy is involved, at least in part, as a cell death mechanism after treatment with ^213^Bi. Nevertheless, when clonogenic colony assays were performed on ^213^Bi-irradiated LP-1 in the presence of 3-MA, cell death occurred to the same extent with or without autophagic inhibition (Figure 8B).

### ^213^Bi-Treated Tumor Cells Activate Dendritic Cells

**Figure 9:**
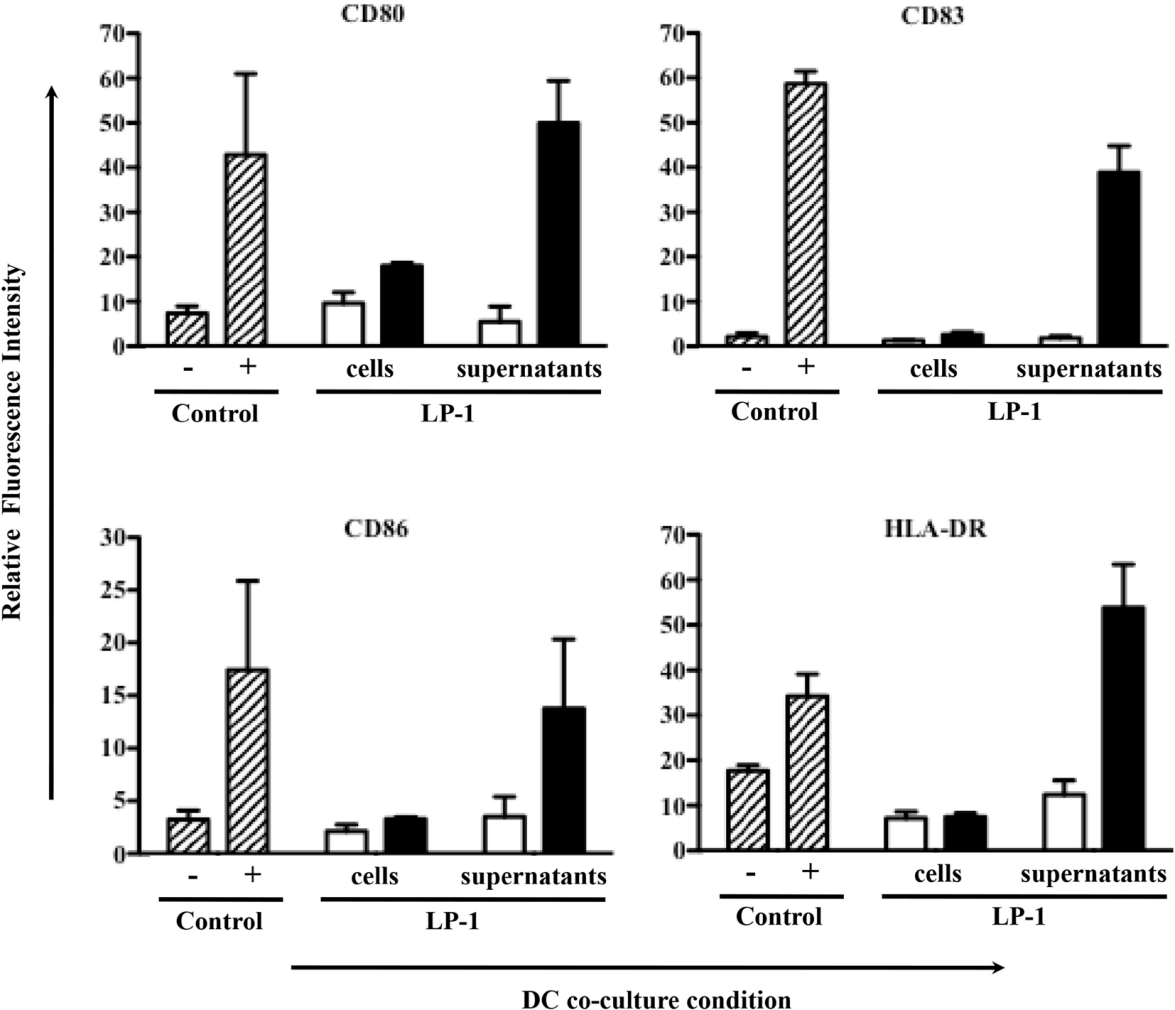
**Effect of ^213^Bi irradiation on immune cells**. DC cell activation markers were analyzed 24 h after coculture with ^213^Bi-treated (black bars) LP-1 cells or LP-1 supernatants as well as untreated (white bars) LP-1 cells or LP-1 supernatants. Control DCs (dashed bars) cocultured alone were used as negative control (−), whereas DCs cocultured with LPS and TNFα were used as positive control (+). Data represent means of ratio of fluorescence intensity ± SEM of two independent experiments.

Besides its direct killing of tumor cells, IR has been shown to induce indirect killing via the immune system. To assess the immunogenicity of alpha-irradiated tumor cells, ^213^Bi-treated LP-1 (425 kBq/mL) or their culture media have been cultured with human DCs. After 1 day of coculture, the DC expression of activation markers (CD80, CD83, CD86, and HLA-DR) was determined by flow cytometry. The culture media of irradiated LP-1 yielded a strong activation of DCs (Figure 9). However, such activation was not observed when DCs were cocultured directly with the irradiated cells alone (without their culture media) (Figure 9). Coculture with irradiated tumor cells plus their culture media did not result in further DC activation (data not shown). These data suggest that the factor(s) capable of activating DCs are secreted in the culture media after irradiation with ^213^Bi. Experiments performed with irradiated 5T33 cells and irradiated 5T33 cell supernatants showed quite similar results on murine DC activation.

To identify the potential factor(s), dosage of common DAMPs was performed on culture media of irradiated 5T33 and LP-1 at 370 kBq/mL or 425 kBq/mL, respectively. No difference in release of HMGB1, Hsp70, or TNFα was observed after irradiation with ^213^Bi (data not shown). The membrane expression of Hsp70, Hsp90, and calreticulin was also tested by flow cytometry; again, no difference was seen after α-irradiation (data not shown).

## Discussion

In this study, we showed that ^213^Bi irradiation of MM cells induced DSBs followed by a cell cycle arrest in G2/M phase and an increased amount of LC3B-II protein, indicating a raise in autophagy. Mechanisms leading to the death of irradiated cells seem to implicate apoptosis, necrosis, and could also involve autophagy.

We used ^213^Bi-BSA, a non-specific vector in order to perform a homogeneous irradiation of the cells. Irradiation was indeed homogenous as demonstrated by the γH2AX flow cytometry histograms, since 100% of cells exhibited DSBs with a maximum reached around 2 h after α-irradiation (Figure 3). Following induction of DSBs by ^213^Bi treatment, we observed a cell cycle blockade in the MM cells. IR is known to induce cell cycle arrest in G1 and G2 phase (24). However, cell cycle blockade in G1 is controlled by p53 and is therefore scarce in tumor cells. It has been shown that α-emitters could also induce a G1 arrest in cells that present a functional p53 (25, 26). The fact that neither LP-1 nor 5T33 cells exhibit such blockade in G1 phase indicates that p53 is deficient in these cell lines. *TP53* gene is actually mutated in LP-1 cells and is also hypermethylated compared to normal plasma cells (27). On the other hand, to our knowledge, no study has shown so far that p53 could be mutated or inactivated in the 5T33 cells. Cell cycle arrest in G2/M phase is common in tumor cell lines and has been shown previously by our team and by others on MM cells (9, 13) and on different types of cancer cell lines (4, 11). This G2/M arrest sensitizes the cells to a second irradiation cycle. Even though they are less dependent than X-rays or γ-radiation, α-particle emitters are not completely insensitive to cell cycle distribution. Indeed, it has been demonstrated that α-irradiation of tumor cells was potentiated when cells were pre-treated with chemotherapies that lead to a blockade in G2 phase (12, 28). These data suggest that combining such chemotherapies with α-RIT or repeated injection of α-RIT could enhance RIT treatment.

When we studied the cell death mechanisms resulting from ^213^Bi irradiation, regardless of the detection method used, we observed only low levels of apoptosis, around 9% and 5% in 5T33 and LP-1, respectively (Figure 5). p53 status in LP-1 and 5T33 cell lines cannot be accounted for this result since both cell lines exhibited very high apoptosis during the same time frame when subjected to UV-B radiation (data not shown). A few studies have shown that tumor cells undergo apoptosis following ^213^Bi irradiation (7–9). These studies used different irradiation protocols, diverse methods to assess cell death, and various tumor cells, which might explain the discrepancy in the results. In particular, Teiluf et al. (9) assessed apoptosis in human MM cells treated *in vitro* with ^213^Bi-anti-CD38 mAb using Western blot analysis which makes difficult to estimate the percent of cells actually undergoing apoptosis. They observed caspase-3 activation and PARP cleavage 48–96 h after irradiation, which correlates with the time frame where we detected some apoptosis in 5T33 and LP-1 cells (Figure 5). Based on their results, we could hypothesize that those cells despite irradiation bypass the G2/M checkpoint to enter mitosis before undergoing apoptosis. This will have to be further studied.

Ionizing radiation induces different cell death mechanisms, including apoptosis, necrosis, necroptosis, mitotic catastrophe, senescence, and autophagic cell death (29–31). We followed cell death over 5 days after ^213^Bi treatment and we observed in between 55 and 95% mortality (Figure 5) while on the long term, clonogenic cell death reached 73.5–88.8% depending on the MM cell line (Table 1). Therefore, other cell death mechanisms might be involved later after irradiation, such as mitotic catastrophe that can occur after several cell divisions or cell senescence that would prevent colony formation. Alternatively, preliminary data in our group showed that expression of DRAM, a gene implicated in autophagy, was increased in T cells after irradiation with ^211^At and ^213^Bi (unpublished data). Therefore, we performed Western blot analyses of LC3B-II form, which is correlated with the presence of autophagosomes at different time points after irradiation with ^213^Bi. Our results show an increase of LC3B-II form after irradiation, and the presence of autophagosomes characterized by a lipidic bilayer membrane was confirmed through TEM. Altogether, these data suggest that α-emitters induce autophagy in MM cells. EBRT is known to induce autophagy (32), and few studies have shown that irradiation with high LET neutrons can also induce such mechanism (33, 34), but to our knowledge, no study so far had investigated the impact of α-particle emitters on autophagy.

The implication of autophagy in cell death has been questioned, being considered either as a programmed cell death mechanism (35, 36) or as a stress response mechanism which can eventually lead to cell death (37, 38). In our study, we observed that the use of autophagy inhibitor 3-MA resulted in protecting LP-1 against ^213^Bi-induced proliferation inhibition, suggesting that autophagy is involved in tumor sensitization to irradiation and cell death induction. Moreover, it has been already shown that excess autophagy could lead to MM cell death (39, 40). We also observed that when clonogenic colony assays were performed on ^213^Bi-irradiated LP-1 in the presence of 3-MA, cell death occurred to the same extent than without autophagic inhibition. This suggests that autophagy is involved in cell death after ^213^Bi irradiation, but when this mechanism is blocked, other cell death pathways are activated, for example, necrosis. However, to reinforce our data, we will have to further depict the exact mechanism of autophagy after ^213^Bi irradiation, by confirming that 3-MA indeed acts on the amount of autophagosomes, by using other inhibitors like bafilomycin A1 that inhibits the final phase of autophagy, the autophagosome degradation, or siRNA directed against protein involved in the autophagic process, and by studying more MM cell lines. It would also be interesting to investigate how cell death occurs when autophagy is inhibited with 3-MA. In this study, we did not observe any effect of 3-MA on the 5T33 cells after irradiation. We used nevertheless the same inhibitor concentration in both MM cell lines (1.25 nM) even though they do not have the same proliferation rate. Indeed 5T33 cells cycle faster than LP-1 cells with a doubling time around 14 versus 28 h, respectively. This cell cycle difference between the two cell lines was also noticeable in proliferation assays where mean ^3^H-thymidine incorporation was around 90,000 cpm for 5T33 at 0 kBq/mL versus around 54,000 cpm for LP-1 (Figure 1) as well as in cell cycle assays where G2/M arrest occurred already at 6 h after irradiation in 5T33 and at 24 h in LP-1 (Figure 4). Therefore, it would be important to repeat the experiments with increasing amounts of 3-MA to ensure that we use the optimal concentration in 5T33 cells.

The role of autophagy in cancer has also been a subject of debate. Some studies have shown that autophagy contributes to tumor cell radioresistance (41, 42) while others have demonstrated that autophagy participates in tumor cell radiosensitization (43, 44). However, a recent study has shown that if autophagy inhibition radiosensitizes tumor cells *in vitro*, it does reduce *in vivo* radioresponse highlighting the importance of the autophagic process in immunogenic signaling, especially through the release of ATP (45). Activation of autophagy is mandatory for ATP secretion in the extracellular medium (46) and leads to ICD (47). Extracellular ATP is a very potent chemoattractant for immune cells to ICD sites (48) and promotes DCs differentiation (49), inflammasome activation, and further proinflammatory IL-1β cytokine secretion (50). In this study, we observed that ^213^Bi-treated MM cells, especially LP-1 cell line, released a soluble factor capable of activating DCs, suggesting that the tumor cells underwent ICD after α-irradiation. We recently demonstrated that ^213^Bi was indeed able of inducing ICD in MC-38 cells resulting in Hsp70 and HMGB1 secretion in the extracellular medium and promoting a specific antitumor immune response (20, 21). However, when we looked at potential DAMPs in ^213^Bi-treated MM cell supernatants, we could not detect any significant release of HMGB1, Hsp70, or TNFα or membrane expression of Hsp70, Hsp90, or calreticulin on irradiated tumor cells. Interestingly, no DC activation was seen when irradiated culture media were subjected to freeze-thaw cycle. This loss of biological activity suggests that the activation factor is unstable but does not provide insights on its molecular nature. Therefore, it would be important to screen other proinflammatory cytokines. Alternatively, based on current literature, it would be interesting to dose ATP or ROS.

In summary, this radiobiology study shows that ^213^Bi induces DSBs in MM cells, followed by cell proliferation arrest related to cell cycle blockade in G2 phase, finally leading to cell death. We only observed low levels of apoptosis, and necrosis seems to be the major cell death mode induced in MM cells after treatment with ^213^Bi. In addition, ^213^Bi irradiation induces autophagy in MM cells, which could be involved in initial proliferation arrest and cell death. Finally, this study opens new prospects on α-particle action since induction of autophagy in MM tumor cells could be an advantageous feature to activate immune cells.

## Author Contributions

J-BG, FD, and JG contributed to conception and design. J-BG, SG, JM, AM, FB, AF-C, YG, MC, and JG contributed to development of methodology. J-BG, SG, JM, AM, FB, AF-C, YG, and JG contributed to acquisition of data. J-BG, SG, JM, AM, FB, AF-C, YG, MC, FD, and JG contributed to analysis and interpretation of data. J-BG, SG, JM, AM, FB, AF-C, YG, MC, FD, and JG contributed to writing and review of the manuscript. MC, FD, and JG contributed to study supervision.

## Conflict of Interest Statement

The authors declare that the research was conducted in the absence of any commercial or financial relationships that could be construed as a potential conflict of interest.
